# *Epilepsia open*—June 2026 Announcements

**DOI:** 10.1002/epi4.70287

**Published:** 2026-06-03

## ILAE CONGRESSES

ILAE Summer School on EEG and Epilepsy

11–19 July 2026

Dianalund, Denmark & Online

1° Curso Latinoamericano de EEG Pediátrico

6–9 August 2026

TBC

16th European Epilepsy Congress

5–9 September 2026

Athens, Greece

16th International Summer School for Neuropathology and Epilepsy Surgery

10–13 September 2026

Erlangen, Germany

16th Asian & Oceanian Epilepsy Congress

19–22 November 2026

Kuala Lumpur, Malaysia

## 2027

16th ILAE School on Pre‐Surgical Evaluation for Epilepsy and Epilepsy Surgery

11–15 January 2027

Brno, Czech Republic

37th International Epilepsy Congress

28 August–1 September 2027

Amsterdam, Netherlands

## WEBINARS

ILAE E‐Forum Series: The role of diagnosis—epilepsy or not, epilepsy type, aetiology

4 June 2026

Consulta de Enfermagem junto à mulher em idade fértil vivendo com epilepsia

30 June 2026

## OTHER CONGRESSES

64th Annual Meeting of the German Society for Epileptology

10–13 June 2026

Würzburg, Germany

Paediatric Epilepsy Training 2—Leeds

11–12 June 2026

Leeds, UK

Paediatric Epilepsy Training 3—Leeds

11–12 June 2026

Leeds, UK

12th Congress of the European Academy of Neurology

27–30 June 2026

Geneva, Switzerland

22nd San Servolo Advanced Epilepsy Course

20–31 July 2026

San Servolo (Venice), Italy

ECON 2026

7–9 August 2026

Ludhiana, India

Epilepsy Imaging in Resource Strained Settings

31 August–12 September 2026

TBC

Workshop on Combined EEG‐fMRI in epilepsy

9–10 September 2026

Athens, Greece

19th Baltic Sea Summer School on Epilepsy

22 September–6 October 2026

Online

2026 CLAE Annual Scientific Meeting

2–4 October 2026

Niagara‐on‐the‐Lake, Ontario, Canada

43rd International Conference on Advances in Psychiatry and Mental Health

5–6 October 2026

Dubai, United Arab Emirates

ILAE British Branch 2026 Annual Scientific Conference

8–10 October 2026

Harrogate, UK

Annual Meeting of the German‐Austrian‐Swiss Epilepsy Working Groups 2026

27–30 October 2026

Vienna, Austria

1st Swiss Neuro Week

18–20 November 2026

Bern, Switzerland

ILAE British Branch Clinical Epilepsy Course for Doctors in Training & Allied Healthcare Professionals

21 November 2026

Birmingham, UK

Encephalitis 2026

7–8 December 2026

London, UK & Online

## 2027

Fourth International congress on Structural Epilepsy & Symptomatic Seizures

7–9 April 2027

Gothenburg, Sweden

